# Anti-GAD65 Antibody-Associated Autoimmune Encephalitis With Predominant Cerebellar Involvement Following Toripalimab Treatment: A Case Report of a Novel irAE of Toripalimab

**DOI:** 10.3389/fimmu.2022.850540

**Published:** 2022-03-25

**Authors:** Huanyu Zhou, Xiaoxi Xie, Tianyu Zhang, Menghan Yang, Dong Zhou, Tianhua Yang

**Affiliations:** ^1^ Department of Neurology, West China Hospital, Sichuan University, Chengdu, China; ^2^ Department of Gastroenterology, West China Hospital, Sichuan University, Chengdu, China

**Keywords:** autoimmune, cerebellum, encephalitis, toripalimab, irAE, anti-GAD65

## Abstract

Toripalimab (Junshi Bioscience Co., Ltd) is a new immune checkpoint inhibitor (ICI) that targets programmed cell death protein 1 (PD-1) in various cancers, including metastatic melanoma. No neurological immune-related adverse events (n-irAEs) of toripalimab have been reported, except for neuromuscular involvement. We report a case of a 63-year-old woman who presented with severe vertigo, vomiting, nystagmus, cerebellar ataxia, and cognitive impairment after toripalimab treatment for metastatic melanoma. Compared with the concomitant cognitive dysfunction and a pathological reflex involving the cerebral cortex, the signs and symptoms of cerebellar involvement were much more prominent. Anti-glutamic acid decarboxylase 65 (anti-GAD65) antibody was positive in both serum and cerebrospinal fluid (CSF). After intravenous immunoglobulin (IVIG) and methylprednisolone (IVMP) administration, the symptoms of vertigo and vomiting resolved, with cognitive impairment and cerebellar ataxia remaining. This is the first report of autoimmune encephalitis (AIE) as an n-irAE of toripalimab.

## Introduction

In the last decade, immune checkpoint inhibitors (ICIs) targeting programmed cell death protein 1 (PD-1) and its ligand PD-L1, as well as cytotoxic T-lymphocyte-associated antigen 4 (CTLA-4), have been a landmark development in cancer treatment, enhancing survival in various cancers by reactivating antitumor immunity. However, ICIs also trigger immune responses against self-antigens, leading to various irAEs, including neurological events. Several n-irAEs have been described in recent years, including encephalitis, myelopathy, aseptic meningitis, meningoradiculitis, Guillain–Barré-like syndrome, peripheral neuropathy, and myasthenic syndrome (1). Most n-irAEs were observed with nivolumab and pembrolizumab, inhibitors of PD-1, and ipilimumab, an inhibitor of CTLA-4. As novel ICIs, n-irAEs related to toripalimab have rarely been reported. Luo et al. first described a neuromuscular triad of myositis, myocarditis, and myasthenia gravis overlap following toripalimab treatment in a patient with metastatic thymoma (2). To date, there have been no other reports on the n-irAEs associated with toripalimab.

Antibodies against glutamic acid decarboxylase (GAD), the rate-limiting enzyme in the synthesis of inhibitory neurotransmitter GABA, are associated with type 1 diabetes mellitus (T1DM) and some neurological disorders, including stiff-person syndrome (SPS), cerebellar ataxia, epilepsy, and limbic encephalitis (3). GAD65-positive neurological irAEs have been observed in several cases, including SPS, cerebellar ataxia, epilepsy, and limbic encephalitis, following ipilimumab and nivolumab treatment (4–6). Herein, we report a case of toripalimab-induced anti-GAD65-associated encephalitis that may expand the irAE profile of toripalimab and provide further experience for clinical oncologists and neurologists.

## Case Presentation

We report a case of a 63-year-old woman with a history of metastatic melanoma who developed severe vertigo and weakness on the day after her first toripalimab injection (3 mg/kg). She was diagnosed with cutaneous left foot melanoma and treated with wide local excision in 2018. Groin and iliac lymph node metastases were detected 20 and 26 months later, respectively, and they were managed with lymph node dissection. The patient received interferon alfa-2b from 2018 to the first dose of toripalimab. The most recent positron emission tomography–CT (PET-CT) before toripalimab administration showed no tumor progression. On the day after toripalimab administration, she developed vertigo triggered by a change in head position, bilateral upper limb tremors, dysarthria, and transient nausea and vomiting for several minutes. The symptoms gradually worsened, and she was confined to the bed. She developed psychiatric disturbances the following week, characterized by confused soliloquy, disorganized thinking, and agitation. The nausea and vomiting worsened after admission.

At admission, neurological examination revealed cognitive impairment (spatial disorientation, memory disorientation, and count disorientation), apparent horizontal nystagmus, ataxia syndrome with bilateral upper limb intentional tremor in the finger–nose test, and right dysmetria in the heel–knee–tibia test. The Babinski sign was positive on the right.

Laboratory studies revealed normal hepatic and renal function, elevated counts of white blood cells and neutral lobulated granulocytes, and a decreased serum potassium level (3.2 mmol/L). Antibodies against Epstein–Barr virus, herpes simplex virus, rubella virus, and cytomegalovirus were negative in the serum. The patient had a history of type 2 diabetes, and her serum glucose level was significantly elevated, ranging from 15 to 25 mmol/L.

Cerebrospinal fluid (CSF) analysis revealed elevated CSF glucose of 6.1 mmol/L (normal 2.5–4.4 mmol/L), elevated protein of 0.49 g/L (normal 0.15–0.45 g/L), normal chloride of 125 mmol/L (normal 120–130 mmol/L), and a cell count of 0 (normal 0–10×10^6^/L). Anti-GAD65 antibody was detected with a titer of 1:30 in CSF and 1:100 in serum, both based on a cell-based assay (CBA). Other autoimmune antibodies against IgLON5, DPPX, GlyR1, DRD2, mGluR5, NMDA, AMPA1, AMPA2, LGI 1, CASPR2, GABAB, mGluR1, amphiphysin, CV2, Hu, Ma1, Ma2, Ri, SOX1, Titin, Tr (DNER), Yo, Zic4, Recoverin, and PKCγ were negative. CSF Gram staining and bacterial and fungal cultures were negative too.

Electroencephalography (EEG) mainly showed a 14- to 20-Hz β-wave. Head magnetic resonance imaging (MRI) with a contrast agent showed no remarkable abnormalities. Chest computed tomography (CT) showed bilateral infiltrates in the lower lobes of the bilateral lungs.

The patient was diagnosed with anti-GAD65-associated autoimmune encephalitis secondary to immune checkpoint inhibitor therapy. Considering the lung infection and hyperglycemia, high-dose glucocorticoid therapy was not considered the first choice. Intravenous immunoglobulin (IVIG) (0.4 mg/kg/day) treatment was initiated in the third week after symptom onset and it lasted for 5 days. After IVIG treatment, the symptoms of vertigo and the psychiatric disturbances resolved. One week after the last IVIG dose, intravenous methylprednisolone (IVMP) (500 mg/day for 5 days) was initiated after the infection, and hyperglycemia was effectively controlled. However, vomiting was not relieved, prompting plasma exchange (PE) 5 days after the last dose of IVMP. The first attempt of PE failed because of a sharp decline in blood pressure, pallor, and tachycardia. We suspected allergic shock related to PE and stopped the PE thereafter. Vomiting symptoms showed gradual alleviation. At discharge, the symptoms of vertigo, vomiting, and weakness had significantly improved, and the psychiatric disturbances had resolved; however, the patient did not achieve complete remission of ataxia and memory disorientation.

**Figure 1:**
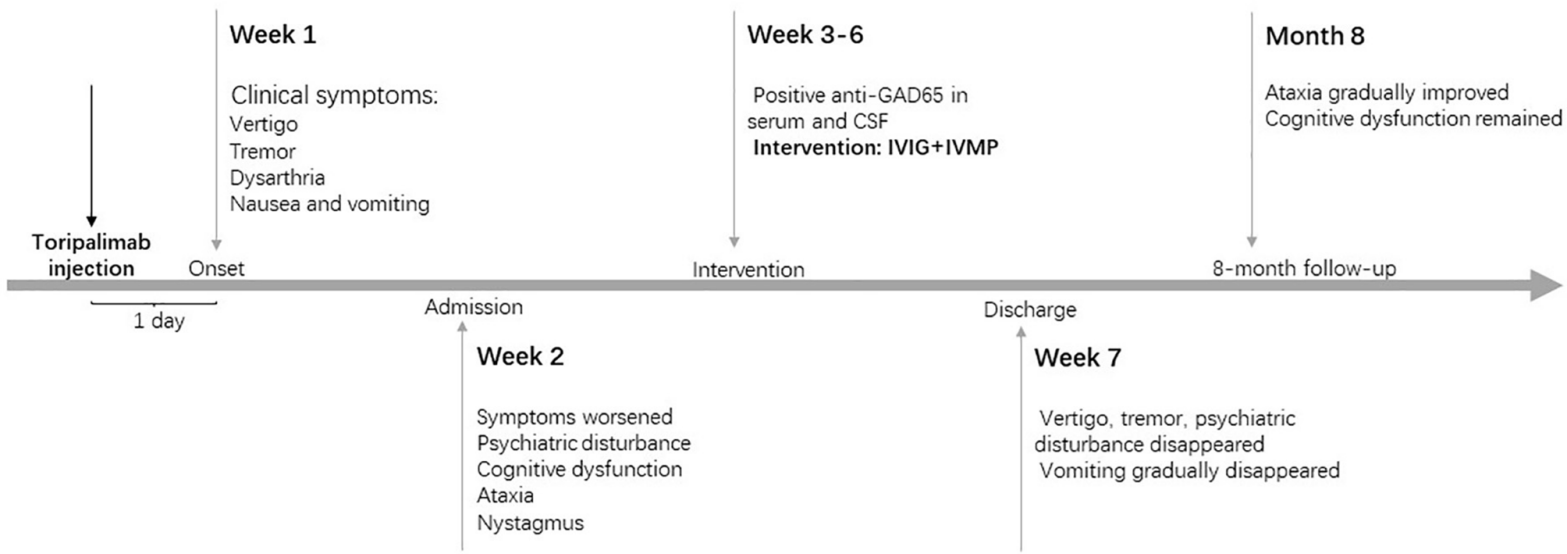
Timeline of patient with relevant data of the episodes and interventions. IVIG, intravenous immunoglobulin; IVMP, intravenous methylprednisolone.

Toripalimab and interferon alfa-2b therapy were discontinued since admission. Eight months later, the patient could stand independently and walk slowly with the support of others, presenting with an ataxic gait with significantly short step length, low step height, and wide support base during walking. Memory dysfunction neither improved nor worsened in the eight months. The word immediate recall test and the word delayed recall test in the Mini-Mental State Examination (MMSE) showed obvious impaired memory function. Repeat head MRI did not reveal any significant changes. Recent computed tomography of the chest and abdomen, as well as lymph node ultrasonography, showed no tumor progression. The clinical manifestations, important results of examinations and related diagnosis and treatments have been organized as a timeline (**Figure 1**).

## Discussion

Compared with myositis and peripheral neuropathies, autoimmune encephalitis (AIE) is not a common n-irAE, but it accounts for more than half of the irAEs in the central nervous system (CNS) (7). The clinical symptoms of autoimmune encephalitis related to ICIs include altered mental status, focal CNS deficits, psychiatric symptoms, seizures, autonomic dysfunction, working memory deficits, ataxia, and dyskinesia (8). According to current studies, most n-irAEs occur early in ICI treatment, usually within 6 months of treatment initiation (9, 10). For most of the reported AIE cases, the first symptoms develop within a mean of 58 days from the first dose of ICIs (nearly 3 cycles of ICI treatment), with a minimum of 3 days (8). The case in our study appears to be the most acute-onset AIE. It is worth noting that cerebellar involvement was prominent, both at onset and at six-month follow-up. Cerebellar ataxia, nystagmus, vertigo, and dysarthria were observed. Immune-induced encephalitis with cerebellar involvement following ICI treatment has been rarely reported. Reina et al. reported a patient presenting with nystagmus and cerebellar ataxia 2 weeks after the initiation of nivolumab therapy for lung adenocarcinoma (11). Acute cerebellitis and corresponding imaging findings of cerebellar edema, early tonsillar herniation, and early hydrocephalus were described in a patient with primary refractory Hodgkin lymphoma being treated with the immune checkpoint inhibitor nivolumab (12). A case of acute cerebellar ataxia due to Epstein–Barr virus (EBV) infection following ICI administration was interpreted as activation of the virus under the affected immune system (13). Autoimmune antibodies, including anti-Zic4, anti-TRIM9, and GAD65, have been detected in some cases (4, 14, 15). Additionally, obvious cognitive impairment supports parenchymal involvement, which is not limited to the cerebellum.

Regarding autoimmune antibodies of AIE related to ICIs, antibodies against intracellular antigens (anti-Ma2, anti-Hu, anti-GAD, an unspecified Purkinje cell antibody, anti-Ri, anti-GFAP) were more frequent than those against cell-surface antigens (anti-NMDA receptor and anti-CASPR2) (4, 6, 8, 16–22). As an autoimmune antibody against intracellular antigens, the anti-GAD65 antibody is associated with several neurological syndromes, including stiff-person syndrome, cerebellar ataxia, and epilepsy (3, 23–25). It is recommended that high serum GAD antibody levels (positive radioimmunoassay or ELISA, positive brain immunostaining, positive cell-based assay, positive line blot) are an important indicator of an immune-mediated cause of the syndrome, and intrathecal synthesis of GAD antibodies provides the strongest evidence that a neurological syndrome is associated with GAD autoimmunity (3, 26). In our case, although we lack evidence of intrathecal synthesis due to a lack of quantitative detection, a positive cell-based assay for anti-GAD65 antibody in both serum and CSF and cerebellar ataxia indicates that the clinical neurological syndrome with anti-GAD antibody in our patient has a probable or definite autoimmune cause, according to the suggested algorithm (3).

Differential diagnoses include paraneoplastic syndrome (PNS) and acute viral encephalitis. First, cerebellar ataxia can occur as a classical paraneoplastic syndrome in patients with or without ICI treatment, always predicting a potential tumor or tumor progression. PNS is not caused by metastatic and/or local effects of cancer on the nervous system; it is instead usually related to cancer-induced immune responses against neuronal proteins (27). For example, paraneoplastic cerebellar degeneration (FCD) can occur several days or weeks after the underlying tumor has been removed and is associated with Yo autoimmune antibodies. Neuroimaging examinations of FCD show normal MRI initially, yet cerebellar atrophy several months later (28). Cerebellar ataxia is associated with anti-Hu, Yo, Tr, SOX1, and VGCC antibodies in some typical tumors such as lung cancer, breast cancer, and Hodgkin lymphoma (28–33). Consistent with other classical phenotypes caused by antitumor immunity (such as limbic encephalitis, peripheral neuropathy, and encephalomyelitis), cerebellar ataxia could also be an irAE of ICIs, in which the anticancer immune response against onconeural antigens is likely to be augmented or even altered under ICI administration because tumors (e.g., melanoma) encounter these phenotypes and positive antibodies are not those tumors (e.g., small cell lung cancer) typically accompanied by PNS (34). We made a diagnosis of n-irAE rather than PNS due to the lack of neurological manifestations before ICI treatment, the acute onset following ICI administration, and the lack of cancer progression in the examinations before ICI treatment (35). In addition, the psychiatric disturbances in the course of the disease and the residual memory impairment may result from inflammation involving the cerebral cortex, which is not reflected on MRI. Approximately 44% of AIEs related to ICIs have negative MRI findings (8). Second, viral encephalitis primarily involving the cerebellum may also present with acute cerebellar ataxia and psychiatric disturbances, usually caused by rotavirus, varicella-zoster virus, Epstein–Barr virus, herpes simplex virus, respiratory syncytial virus, mumps virus, parvovirus B19, and other rare virus types. Autoimmune antibody-mediated encephalitis may also result from postinfectious autoimmunity. However, viral CNS infections always have distinctive CSF changes and elevated cell counts, and viral encephalitis always has precursor viral infectious symptoms. Despite the lack of nucleic acid detection of the virus in CSF, negative results of antibodies against Epstein–Barr virus and herpes simplex virus could also help in differentiation.

Encephalitis has the second-highest mortality rate after myasthenia gravis and a relatively lower improvement rate in n-irAEs (7, 10). According to the guidelines, pulse corticosteroids, methylprednisolone, and IVIG are recommended (36). If autoimmune antibodies are positive and there is limited or no improvement, rituximab or plasmapheresis is considered (36). Patients without focal syndromes and those with negative antibody focal syndromes have a good prognosis, and compared with other autoantibodies, those with anti-GAD antibody or anti-cell-surface antibodies also have a favorable prognosis (37). Additionally, abnormal MRI findings are associated with poor outcomes, and prolonged cognitive deficits were observed in 79% of patients with AIE (8, 37). In our case, although cognitive impairment and ataxia gait did not achieve complete remission after first-line immunotherapy, the patient’s symptoms significantly improved.

## Conclusion

In this report, we describe a patient with metastatic melanoma who developed anti-GAD65-associated autoimmune encephalitis with predominant cerebellar involvement following toripalimab treatment and achieved some improvements after immunoglobulin and methylprednisolone therapy. As toripalimab is a new ICI targeting PD-1, its related irAEs have scarcely been reported, let alone its n-irAEs. We have provided a novel experience regarding the n-irAEs of toripalimab.

## Patient Perspective

Although the patient was still staggering when walking and could not think quickly, she thought the treatment was effective. It took the excruciating vertigo and vomiting away, which freed her from being completely dependent on others for help.

## Data Availability Statement

The original contributions presented in the study are included in the article/supplementary material. Further inquiries can be directed to the corresponding authors.

## Ethics Statement

The studies involving human participants were reviewed and approved by Ethics Committee on Biomedical Research, West China Hospital of Sichuan University. The patients/participants provided their written informed consent to participate in this study.

## Author Contributions

HZ and TY initiated the study and wrote the manuscript. XX, TZ, and MY collected clinical data. DZ and TY reviewed the manuscript. All authors contributed to the article and approved the submitted version.

## Funding

The study was supported by the major Program of Science and Technology Commission Foundation of Sichuan Province (grant number: 2020YFG0078).

## Conflict of Interest

The authors declare that the research was conducted in the absence of any commercial or financial relationships that could be construed as a potential conflict of interest.

## Publisher’s Note

All claims expressed in this article are solely those of the authors and do not necessarily represent those of their affiliated organizations, or those of the publisher, the editors and the reviewers. Any product that may be evaluated in this article, or claim that may be made by its manufacturer, is not guaranteed or endorsed by the publisher.
